# The data transparency crisis in research: Lessons from systematic reviews and meta-analyses

**DOI:** 10.1371/journal.pmed.1005145

**Published:** 2026-06-16

**Authors:** Saul Martin-Rodriguez, Rodrigo Fernandez-Gonzalo, David Moher

**Affiliations:** 1 Department of Laboratory Medicine, Division of Clinical Physiology, Karolinska Institutet, Stockholm, Sweden; 2 Unit of Clinical Physiology, Karolinska University Hospital, Stockholm, Sweden; 3 Centre of Journalogy, Methodological and Implementation Research Program, Ottawa Hospital Research Institute, Ottawa, Ontario, Canada; 4 Institute of Health Policy, Management & Evaluation, Dalla Lana School of Public Health, University of Toronto, Toronto, Ontario, Canada

## Abstract

- Systematic reviews and meta-analyses underpin clinical guidelines and health policy, yet their validity may be compromised by limited access to underlying datasets and associated analytical code.
- Reliance on incomplete or inconsistently reported summary statistics forces researchers to use imputation and unverifiable assumptions, which can distort effect estimates and mislead clinical decision-making.
- The consequences extend beyond methodology: flawed evidence synthesis can influence treatment recommendations, healthcare spending, and patient safety, as illustrated by historical cases such as hormone replacement therapy.
- Despite widespread data-sharing policies, compliance remains low, enforcement weak, and monitoring almost non-existent, with many datasets remaining unavailable or inaccessible.
- This Policy Forum argues for strengthening enforceable data-sharing mechanisms, including clearer enforcement and pragmatic verification approaches within editorial workflows.

## Abstract 2

Systematic reviews and meta-analyses underpin clinical guidelines and health policy, yet their validity may be compromised by limited access to underlying datasets and associated analytical code. This Policy Forum by Saul Martin-Rodriguez and colleagues argues for strengthening enforceable data-sharing mechanisms, including clearer enforcement and pragmatic verification approaches within editorial workflows.

## Introduction

Systematic reviews and meta-analyses (SRMAs) constitute the backbone of evidence-based medicine, shaping clinical practice guidelines, regulatory policies, and treatment protocols. However, important concerns have been raised regarding their reproducibility and transparency, particularly due to a critical and pervasive obstacle: limited access to the underlying data and analytic code. The inability to obtain raw data from the underlying primary studies, whether at the level of individual participant data or structured study-level datasets, often forces meta-analysts to rely on aggregate data and summary statistics, which may be inconsistently reported and require imputation. These limitations may affect the robustness and interpretability of SRMAs, with potential implications for clinical decision-making. Although these challenges are often most visible in the context of clinical trials, similar issues arise across a wide range of research designs, including observational studies and other forms of data-driven research.

Despite the increasing adoption of data-sharing policies, their implementation remains suboptimal. Data-sharing practices may also vary across study designs, with clinical trials often subject to more formalized sharing requirements than observational or qualitative research, although compliance remains inconsistent. A study analyzing 7,750 biomedical research papers published between 2015 and 2020 found that only 9% of all papers pledged to make their data publicly available, and a mere 3% of the total followed through with this commitment [1]. Even in journals with explicit data-sharing mandates, enforcement remains weak. In a study analyzing 3,556 articles, 3,416 included a data availability statement (DAS). Of these, 42% indicated that data were available upon request, which does not necessarily constitute effective or immediate data sharing. Among the 1,792 manuscripts whose DAS explicitly pledged data sharing, 1,669 authors (93%) either did not respond or declined to provide their data. Consequently, only 123 authors (6.8%) ultimately supplied the requested datasets [2]. The lack of adherence to data-sharing policies, whether mandated by journals, funders, regulatory bodies, or research-performing organizations (RPOs), represents a substantial barrier to scientific integrity, reinforcing the need for enforceable transparency measures. As has been argued, the absence of full access to clinical trial data has historically allowed distorted evidence to guide widespread treatment decisions, exposing patients to unnecessary risks and undermining trust in science [3]. For those conducting systematic reviews, the practical burden is considerable: requests for data often go unanswered for weeks or months, and in many cases, no response is received. This lack of responsiveness makes it difficult to determine whether barriers are logistical, institutional, due to perspectives of the primary researchers, or related to broader concerns such as data governance, ownership of the data, or selective reporting.

In many settings, legal and regulatory constraints imposed by research ethics committees, governments, or data governance frameworks may restrict data sharing, particularly across jurisdictions or in the context of large-scale cohort studies and biobanks [4,5]. Ethical considerations related to participant privacy and consent may further limit the extent to which underlying data can be made publicly available. In addition, incentive-related factors, including concerns about data ownership, future use of datasets, and potential scrutiny of analyses, may also discourage researchers from sharing data and code. Thus, limited data availability often reflects a complex interplay of structural, ethical, and cultural constraints rather than a simple lack of willingness to share.

The consequences extend beyond academic debates and into real-world clinical applications. Limitations in data accessibility may affect the reliability of the evidence base used to inform practice and policy across a wide range of conditions. Ultimately, the absence of accessible underlying data and code contributes to research inefficiencies, with multiple groups attempting to replicate work that could have been verified, thereby increasing research waste and limiting the full potential of SRMAs.

## The unseen bottleneck: Data availability and meta-analysis validity

The reliability of an SRMA hinges on the quality and completeness of the data it synthesizes. Yet, many primary studies fail to provide essential data in accessible, standardized formats. Key findings are often presented as quartiles, interquartile ranges, or median values, rather than means and standard deviations, which can complicate the calculation of standardized effect sizes and introduce additional methodological uncertainty [6]. Established methods can be used to perform these conversions when appropriately specified and reported [7–9]. While this is unavoidable for inherently non-parametric or binary variables, in many cases, such formats reflect reporting conventions rather than measurement constraints and may limit the ability to extract comparable effect estimates required for quantitative synthesis. This limitation is context-dependent and does not apply equally across all types of outcomes. Even more problematic, many primary studies omit essential covariate-level data, limiting the scope of advanced approaches such as meta-regression.

Despite the clear need for complete, standardized datasets, missing information remains a persistent obstacle, particularly in approaches such as multiple regression meta-analyses, where comprehensive covariate data are crucial. Researchers often resort to imputation methods, including the assumption or estimation of pre–post correlation coefficients, to fill these gaps; however, even when sensitivity analyses are conducted, such approaches may introduce additional uncertainty and potential bias [10]. When multiple studies require imputation, cumulative errors can propagate across analyses, and recent investigations have shown that imputing missing correlation coefficients can substantially alter measures of heterogeneity and even change the conclusions drawn from the data [10]. The presence of incomplete datasets thus may affect both the precision and interpretability of SRMA findings, with potential downstream implications for clinical and policy decision-making. Importantly, variability in analytical results is not inherently problematic, as different analysts may reasonably reach different conclusions when applying valid but distinct methodological choices [11]. However, limited access to underlying data and code restricts the ability to interrogate, reproduce, and contextualize such differences.

From a policy perspective, these miscalculations can extend beyond academic interpretation and into real-world decision-making. Government agencies and public health bodies frequently base reimbursement and coverage decisions on meta-analytic findings, effectively shaping healthcare resource allocation and, in some cases, entire markets [12]. For example, according to the Institute for Clinical and Economic Review, unsupported price increases in selected US drugs without new evidence of clinical benefit added an estimated US$ 815 million in 2023 spending [13]. Consequently, if the underlying evidence base overestimates or underestimates intervention effects, this may contribute to suboptimal allocation of public resources, while potentially overlooking more effective therapies.

Incomplete or inconsistently interpreted evidence can further influence regulatory decisions, prescribing practices, and public perception, as illustrated by longstanding debates surrounding the safety profile of statin therapy [14,15]. In this regard, disagreement over the frequency and clinical relevance of statin-associated adverse effects has contributed to conflicting interpretations of the evidence, influencing both clinical practice and public confidence in treatment recommendations. Strengthening data and code transparency may improve the robustness and reproducibility of SRMA, supporting more reliable decision-making.

## The consequences of flawed meta-analyses in clinical practice

What happens to clinical knowledge when no system exists to ensure that underlying data remain accessible beyond the published record? A striking historical example illustrates the issue. For nearly two decades, aggregate-data meta-analyses of observational studies suggested that menopausal hormone therapy (HRT) reduced coronary heart disease (CHD) risk by almost 50% in relative terms, a conclusion that shaped clinical guidelines worldwide and influenced the care of tens of millions of women. These syntheses, based solely on published summary statistics, were incapable of adequately adjusting for confounding or examining effect modification, yet they became an authoritative evidence base. In the most influential meta-analysis of the period, the pooled relative risk for CHD among HRT users was 0.56 (95% CI 0.50–0.61), reinforcing the narrative of cardioprotection [16]. This narrative collapsed only when detailed participant-level data from the Women’s Health Initiative (WHI) became accessible. With full access to adjudicated outcomes, covariates, and time-to-event information, the WHI randomized trial revealed that combined estrogen plus progestin therapy increased risks of coronary events, stroke, pulmonary embolism, and breast cancer. This reversal was so dramatic that the trial was halted early for harm [17]. Subsequent analyses integrating extended follow-up confirmed persistent excess risks and demonstrated that age- and time-since-menopause effects required analysis at the participant-level [18]. These findings contributed to major changes in clinical guidelines, prescribing patterns, and regulatory policies in multiple countries.

Crucially, this event was not a case of unpublished studies or selective reporting. Every major observational study was publicly available. What was missing were the underlying datasets necessary to detect confounding, test competing models, and evaluate subgroup effects. Working solely with published summary statistics, meta-analysts produced confident and reproducible estimates that were later shown to be inconsistent with evidence from randomized trials, shaping treatment decisions for decades. Earlier access to underlying datasets may have facilitated more robust evaluation of confounding and subgroup effects, potentially reducing the risk of widespread misinterpretation and its downstream clinical and policy consequences. HRT became one of the most widely prescribed therapies in high-income countries during the 1990s, with annual expenditures reaching into the billions of dollars. When WHI results emerged, prescribing rates collapsed within months, exposing the extent to which incomplete data had misled clinicians, regulators, and policymakers. These events highlight how earlier access to transparent, analyzable datasets may have enabled more robust evaluation of confounding and effect modification, potentially influencing how the cardioprotective narrative was interpreted and adopted in practice.

The HRT episode is not a historical anomaly but an emblem of a systemic weakness in how evidence is generated, reported, and synthesized. When access to underlying data is limited, the capacity of meta-analyses to provide a clear and reliable synthesis of evidence may be compromised. Evidence from data-sharing initiatives reinforces this structural point. In a review of 37 published reanalyzes based on shared clinical trial datasets, 13 reached interpretations that differed from those of the original publications, illustrating how access to underlying data can materially alter scientific conclusions and prevent the persistence of inaccurate findings [19], extending beyond academic interpretation, with implications for patient outcomes, public trust, and healthcare resource allocation.

## Towards practical and enforceable data transparency

**Fig 1:**
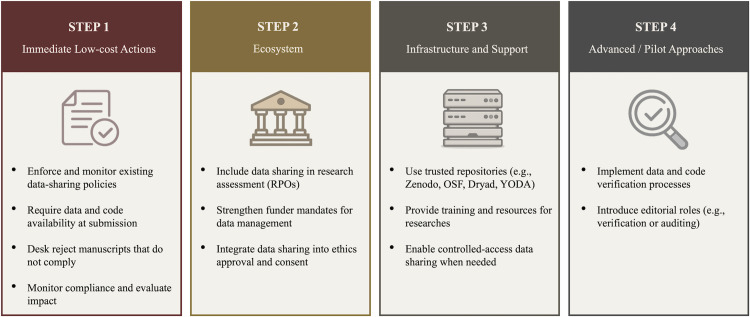
A stepwise framework for improving data transparency in research. Schematic representation of a pragmatic, multi-level framework to enhance data and code availability in research. The model outlines progressive steps ranging from immediate, low-cost actions (e.g., enforcement of existing policies) to broader system-level alignment, infrastructure development, and the potential implementation of advanced verification mechanisms. The framework emphasizes feasibility, scalability, and coordinated action across journals, funders, and research-performing organizations. Illustrative icons included were generated using ChatGPT (OpenAI, San Francisco, CA, USA) image generation tools and subsequently adapted and integrated into the final figure by the authors.

Improving data transparency in research requires a shift from policy statements to consistent, enforceable, and monitored practices (Fig 1). Although many journals and funders mandate data-sharing statements, compliance remains inconsistent, enforcement limited [2], and public-facing monitoring non-existent. A practical first step is the more consistent application of existing requirements, including making data and code available at the time of publication for all research whose findings are based on data.

Authors should be encouraged, and where feasible required, to deposit analysis-ready datasets and associated code in recognized and trustworthy repositories (e.g., Zenodo, Open Science Framework, Dryad, or domain-specific platforms), as a condition of publication, unless clear legal or ethical constraints apply [3]. Data sharing should include sufficient documentation and metadata to enable meaningful interpretation and reuse, recognizing that this requires appropriate support and domain-specific expertise [20]. Journals could adopt clearer and more enforceable and monitored policies, including, where appropriate, desk rejection of manuscripts that do not comply with data availability requirements, except where legitimate constraints apply. In such cases, authors should provide transparent justification and facilitate controlled access to data. Data sharing should follow the principle of being “as open as possible, and as restricted as necessary” [20].

It is also important to consider equity in the implementation of data-sharing requirements. Researchers working in low- and middle-income countries or in resource-constrained settings may face additional barriers to data sharing [21,22]. Rigid mandates risk excluding valuable evidence from certain regions. To minimize this risk, policies should adopt flexible, principle-based approaches, be supported by appropriate infrastructure and training, and facilitate access to suitable data repositories. Such measures are essential to ensure that efforts to improve transparency do not exacerbate existing global inequalities in research.

Beyond journals, RPOs and funders have a critical role to play. Recent work has highlighted the need for RPOs to take a more active role in shaping research quality, transparency, and trustworthiness through institutional policies and assessment frameworks [23]. In this context, data and code sharing should be explicitly recognized as core components of responsible research assessment, alongside more traditional metrics. Embedding data-sharing expectations early in the research process (e.g., ethics approvals and consent procedures) and providing dedicated support for data management may facilitate implementation. Evidence suggests that many research participants support data sharing when appropriate safeguards are in place, highlighting the importance of integrating transparent data-sharing provisions within informed consent processes [24,25]. Emerging work further emphasizes that research ethics boards play a critical role in enabling data sharing by ensuring that consent language explicitly accommodates data deposit and reuse, as consent fundamentally determines the downstream conditions under which data can be accessed and shared [26].

Importantly, access to data alone may be insufficient without the accompanying analytical code required to reproduce and meaningfully interrogate findings [27,28]. More resource-intensive approaches may also be considered. For example, journals could explore the introduction of dedicated roles or processes to verify data and code availability, ensuring that deposited materials are accessible and sufficiently documented to support reproducibility. However, such approaches would require careful evaluation of feasibility, cost, and scalability, and may be best implemented initially through targeted pilot studies within selected journals. Verification mechanisms should be viewed as complementary to, rather than a substitute for, stronger policy enforcement.

Finally, any strategy to improve data transparency must include mechanisms for monitoring and evaluation. Progress is likely to be gradual and context-dependent, requiring coordination between journals, funders, institutions, and the broader research community.

## Conclusions

The persistent gap between data-sharing policies and their implementation continues to limit the reproducibility and interpretability of research findings. Addressing this gap requires moving beyond aspirational statements towards consistent and enforceable practices across journals, funders, and RPOs.

“Extraordinary claims require extraordinary evidence”. Nearly five decades after Carl Sagan articulated this principle, it remains highly relevant: scientific claims are still often disseminated without access to the underlying data and code needed to evaluate them.

Progress, however, is achievable. Greater coordination across journals, funders, regulators, and institutions can help make data and code availability standard practice, fostering a research culture in which transparency and reproducibility are embedded in everyday scientific work.

## Abbreviations

CHD: coronary heart disease
DAS: data availability statement
HRT: hormone therapy
RPOs: research-performing organizations
SRMAs: systematic reviews and meta-analyses
WHI: Women’s Health Initiative
